# India’s Traditional Knowledge Digital Library and the Politics of Patent Classifications

**DOI:** 10.1007/s10978-021-09299-7

**Published:** 2021-06-12

**Authors:** Martin Fredriksson

**Affiliations:** Department of Culture and Society, Linköping University, Linköping, Sweden

**Keywords:** India, Patent classification, Patent law, Traditional knowledge, Traditional knowledge digital library

## Abstract

This article analyzes India’s Traditional Knowledge Digital Library (TKDL) as a potential intervention in the administration of patent law. The TKDL is a database including a vast body of traditional medical knowledge from India, aiming to prevent the patenting and misappropriation of that knowledge. This article contextualizes the TKDL in relation to documentation theory as well as to existing research on the uses of databases to protect traditional knowledge. It explores the TKDL’s potential consequences for India’s traditional medical knowledge and the wider implications that traditional knowledge databases can have for the safeguarding of traditional knowledge in general. The article concludes that on the one hand the TKDL bridges the gap between the main branches of Indian traditional medicine and the formal knowledge system of International Patent Classifications. Furthermore, it has also inspired revisions of the International Patent Classification system, which makes it better adapted to incorporate traditional medical knowledge. On the other hand, critical research on traditional knowledge documentation argues that traditional knowledge databases, like the TKDL, can decontextualize the knowledge they catalogue and dispossess its original owners. The TKDL, however, also fits into a national, Indian agenda of documenting and modernizing traditional medicine that predates the formation of the TKDL by several decades and challenges the dichotomy between traditional and scientific knowledge systems that originally motivated the formation of the TKDL.

## Introduction

Classification systems of all types are at base social institutions that reflect and describe how things are in the social world. (Bowker and Star 1999, p. 61)

Bowker and Star’s notion that classification systems reflect social relations applies to a wide range of areas, from medical diagnostics to the categorization of science and knowledge. The social significance of classification implies that changes to classification systems can also have consequences for the social world and the power relations it entails. The relation between classification and power is particularly evident in patent law, a field where property rights and the classification and organisation of information intersect. While patents have been thoroughly investigated and debated as property, they are rarely contextualized in relation to documentation studies (Hemmungs Wirtén 2019). This article approaches the administration of patents as a classification system with profound implications for the circulation of information in general, and the protection and misappropriation of traditional knowledge in particular. It takes India’s Traditional Knowledge Digital Library (TKDL) as a case study to discuss the implications of traditional knowledge databases as a potential social justice intervention.

The TKDL is a database that collects and catalogues information on practices and pharmaceutical formulas originating from the four main branches of traditional Indian medicine: Ayurveda, Siddha, Unani and Yoga. The TKDL was founded in 2001, but its prehistory can be traced back to two infamous patent cases from the 1990s: the turmeric case and the neem case. In 1995, two scientists from the University of Mississippi were granted a patent on the uses of turmeric, *Curcuma longa*, to heal wounds. Two years later, the patent was revoked by the US Patent and Trademark Office after objections from India’s Council of Scientific and Industrial Research (CSIR), which claimed that it relied on traditional practices that had been known in India for centuries. During the same period the American chemical company W.R. Grace had, along with a number of other international corporations, been granted patents on various uses of the substance Azadirachtin. Since Azadirachtin was derived from the neem tree, which has been used in numerous ways by generations of Indian farmers, a coalition of Indian and international NGOs challenged the patent, which was finally revoked by the European Patent Office (EPO) in 2000 (Reddy and Chandrashekaran 2017; Dutfield and Suthersanen 2019; Neem Foundation 2021).

These cases were of strong symbolic importance, both in India and globally, as they evoked a history of colonial exploitation of traditional knowledge. In the 1990s a discourse about so-called biopiracy had been growing, criticizing the illegitimate appropriation of locally held traditional knowledge by non-local commercial actors. Biopiracy was usually associated with western biotech companies who forage the fauna of biodiversity-rich developing countries to exploit and commercialize biological substances that have been used by local people for generations (Shiva 1997; Robinson 2010; Robinson et al. 2014; Fredriksson 2017). Since the 1990s, biopiracy and the protection of local, traditional knowledge has, as Chidi Oguamanam (2008, p. 32) puts it, become ‘a touchstone for solidarity among indigenous peoples and former colonies of the south […] in their resistance to Western knowledge hegemony, or what Drahos and Braithwaite call information feudalism’.

Like many other biodiversity-rich countries in the global south, India had seen its traditional knowledge appropriated by foreign companies for decades. Unlike most developing countries, India has, however, been known to take an openly defiant stance towards attempts from the global north to impose its patent agenda in India (Reddy and Chandrashekaran 2017). Foreign exploitation of India’s traditional knowledge has been addressed in national legislation such as the Indian Patents Act from 1970 and its 2002 amendments, but India has also tried to influence international regulation within the UN and the World Trade Organization (Sen and Chakraborty 2014, p. 1341). Despite these efforts, biopiracy and misappropriation of Indian traditional medicine has persisted, not least in relation to traditional medical knowledge. Even though some argue that the neem and turmeric cases had limited practical consequences for India (Reddy and Chandrashekaran 2017; Dutfield and Suthersanen 2019), they tapped into this existing discontent over decades of colonial exploitation and triggered what Reddy and Chandrashekaran (2017, p. 270) call ‘a misguided sense of wounded Indian pride’. Along with a range of previous cases of alleged misappropriation of traditional knowledge from India, the neem and turmeric cases came to mobilize a political initiative to address the problem of foreign appropriations of Indian cultural heritage in a more systematic way.

Dutfield and Suthersanen (2019, p. 16) argue that particularly the neem case had a strong impact on international discourse as it ‘reinforced the classical biopiracy narrative which depicted corporations, typically from the United States, as preying on the peoples of the developing world to steal their knowledge’ and shaped international debates on benefit sharing within the UN and World Trade Organization. The turmeric case, on the other hand, came to be more formative for the TKDL as it implied that the problem of misappropriation was, at least partly, caused by different ways of documenting and classifying knowledge. Even though the uses of turmeric were known in every Indian household, the patent was revoked on the grounds of existing prior art only when the CSIR could provide evidence that this knowledge had been documented in ancient Sanskrit texts and in a scientific paper from 1953 (Suwapan 2016). While the Patent Cooperation Treaty (PCT, articles 15 & 16) requires that all patent applications shall be subjected to an international search for prior art, the sources are generally assumed to be written or visual documentation. Moreover, these sources must be reasonably accessible to patent examiners, which rules out rare publications that can only be found in languages and libraries that are inaccessible to most patent examiners (WIPO/GRTKF/IC/5/6, §6). So, even though CSIR eventually managed to produce written documentation of the prior use of turmeric, that documentation was not of the kind that the patent examiners could have been expected to find of their own accord.

The turmeric case thus spoke to the needs to document India’s traditional knowledge in ways that make it identifiable as prior art by foreign patent examiners (Suwapan 2016). In September 1999, the Department of Indian Systems of Medicine and Homeopathy addressed this problem by appointing an interdisciplinary *Task Force on Conservation and Sustainable Use of Medical Plants* (Reddy and Chandrashekaran 2017). In November 2000 the Task Force published what can be described as a position paper where it argued for the need to create a digital library of Indian traditional medical knowledge, followed by a report in December 2000, which presented the outlines of a Traditional Knowledge Digital Library (Gupta 2000, 2001). This report explicitly refers to the turmeric and the neem cases, arguing that although India eventually had those patents invalidated, it took effort and resources. The report concluded that it would be better to take a preemptive approach and create a database that would prevent the issuing of bad patents, instead of going through the hurdles of challenging them retrospectively (Gupta 2001). Consequently, the TKDL was established in 2001 as a catalogue on prior art that could help patent examiners to identify and reject patent applications based on already existing Indian traditional medical knowledge.

At an early stage, WIPO’s *Intergovernmental Committee on Intellectual Property and Genetic Resources, Traditional Knowledge and Folklore*, also founded in 2001, endorsed the TKDL as a good model for traditional knowledge protection (Fredriksson forthcoming). The TKDL was thus, already from the outset, framed as part of a wider movement to challenge a postcolonial order of power and knowledge and protect traditional knowledge originating from indigenous people and developing nations against exploitation from industrialized countries and multinational corporations. This makes the TKDL a textbook example not only of classification systems as social institutions, but more specifically of the traditional knowledge database as a global social justice institution. This article takes the TKDL as a case study to discuss the wider implications that traditional knowledge databases can have for the safeguarding and circulation of traditional knowledge.

Methodologically, the article combines a close reading of reports, protocols and other documents regarding the TKDL with secondary sources that describe its origins and design. It analyses these documents and sources in light of theories of documentation and in relation to recent research on the protection and documentation of traditional knowledge. The article sets out with a description of the classification system of the TKDL and its integration in the International Patent Classification system. This is followed by a discussion where the TKDL is related to a body of critical research on how traditional knowledge documentation affects the knowledge it documents. Finally these empirical and theoretical accounts are related to the role the TKDL plays in a national, Indian, context which partly challenges the framing of the database as part of an international movement to protect the rights of indigenous people and developing countries.

## Classifying Traditional Medicine

In the position paper from November 2000, V.K. Gupta—spokesperson for the *Task Force on Conservation and Sustainable Use of Medical Plants* and the TKDL’s first director—explains why India needs a traditional knowledge database. Gupta (2000, p. 307) begins by stating that: ‘Globally, there are two distinct and potentially conflicting knowledge systems. The knowledge system of the formal sector, of both private and public institutions, and the knowledge system﻿ of the informal sector of communities and individuals.’ While the former is well documented, institutionalized and often protected by intellectual property rights, the latter is largely oral, mostly undocumented and often hard to protect.

As the TKDL came to demonstrate, these systems are not incommensurable, but bridging the gap between them calls for new approaches to classification. One of the architects behind the TKDL explains: ‘if you want patent examiners to take this prior knowledge into account when looking at applications claiming the novelty of therapeutic formulations, you have to make it comprehensible, you have to translate it into a language they understand’ (Gaudillière 2014, p. 392). This was in line with the position of the World Intellectual Property Organization (WIPO), which also concluded that the misappropriation of traditional knowledge was partly caused by patent examiners’ inability to identify ‘relevant traditional knowledge as prior art’ and called for initiatives that could bridge the gap between patent offices and traditional knowledge documentation (WIPO/GRTKF/IC/2/6). Consequently, the TKDL needed to provide a translation not only between languages, but also between knowledge systems and make traditional medical knowledge available in a format that is accessible to patent examiners across the world, or as Gupta puts it: to prepare a ‘format to suit’ WIPO (Gupta 2001, p. 122). The first step towards creating the TKDL was thus to build the architecture and the software for sorting and storing the data in a searchable format. This was done through the so-called Traditional Knowledge Resource Classification system (TKRC), which provides a language-independent format especially suited for categorizing metadata about traditional medical knowledge. Once this was in place a team of 30 Ayurvedic experts, five IT experts, four scientists and two patent examiners began collecting, translating and classifying content sourced from a wide range of texts on traditional Indian medicine (WIPO/IPC/CE/31/6). A large number of traditional formulas were codified and by March 2003 a first batch of data was in place. Six months later a demo of the TKDL was released (CSIR 2021).

The TKRC was created specifically for the TKDL, but it was not created without precedence as it had to relate to existing standards of classification that govern the global patent systems. The position paper compares the TKDL to WIPO’s Intellectual Property Digital Library (IPDL)—an electronic catalogue of data on patents and trademarks that was also under construction at the time (WIPO/SCIT/5/5)—but with the major difference that the IPDL contains information on already existing patents, while the TKDL collects information on non-patented knowledge that can serve as prior art (Gupta 2000). Gupta concludes that the ‘[f]irst level of search in an IP office begins with the sheet of the patent application, therefore it may be prudent to create TKDL based on information similar to that of the first sheet of a patent document’ (Gupta 2000, p. 310). So, even though the content of the TKDL is not patented innovations, it is best presented in a way that mimics that of patents. Consequently, the posts in the TKRC were described according to key attributes taken from the IPDL, such as ‘title, knowledge resource, date since known, country, contact organization, abstract on usage, key words, IPC’ (Gupta 2000, p. 311).

The last key attribute—IPC—refers to the classification code provided by the International Patent Classification system (IPC): a documentation system launched by WIPO in 1971 to make patents internationally searchable (Kang 2012; WIPO 2019). In order to make its content easily accessible all over the world, TKRC based its classification system on the IPC (Gupta 2001; Thomas 2010; Sen and Chakraborty 2014; Suwapan 2016; Anilkumar 2018). The IPC is a hierarchical classification system that categorizes all kinds of inventions into five levels of subdivisions defined by a combination of letters and numbers. The classification begins with a single letter—from A to H which represents a section. This is followed by a two digit number representing a class; a single letter representing a sub-class; a one to three digit number representing a group and finally a two to five digit number representing a sub group (Adams 2000). The result is a system where all different kinds of inventions—from hat pins (A44B 9/06) to driving mechanisms for harvesters (A01D 69/00)—are classified into subgroups such as A61K 31/43 (medicinal preparations containing penicillin). The 2020 edition of the IPC contained approximately 70,000 such subgroups. The classification system is maintained by WIPO and continuously updated by the IPC committee of experts. While the signatory states are required to adapt their national patent documentation to the IPC system, they are also invited to take part in the annual revision of the classification system (Kang 2012). The TKRC adopted the same system of classification as the IPC, but developed its own set of categories that were particularly suited to its specific content. Consequently, India’s traditional medical knowledge was categorized into 25,000 subgroups that were compatible with the IPC (CSIR 2021).

Jean Paul Gaudillière (2014, p. 399) describes how TKDL draws its content from a large body of ancient texts and documents that are being deciphered, translated and classified by a group of, mostly female, practitioners of Ayurveda, Siddha, Unani and Yoga:

each of them typing in information on a classical formulation read in Sanskrit texts and using a whole set of English, Hindi, Malayalam, and Urdu dictionaries. The translations they were crafting supposed a set of scarcely obvious equivalences between the vernacular denominations of medical materials and the ‘modern’ botanical denominations, on the one hand, or between the etiological and nosological categories of biomedicine and those of the Indian medical system considered, on the other.

A standardized form was visible on each screen […], displaying the basic structure that had been adopted for the database. Coded in numbers defined in a new general classification scheme […] The final product, as displayed on the TKDL website, is a form taking the formulation as a unit but presenting it in a format any patent-document reader will find familiar.

Gaudillière’s account gives a glimpse of how this traditional medical knowledge is processed through the TKRC, in ways that translate and fit that knowledge into a format—a documentation template—which is prescribed by WIPO and the IPC.

According to the CSIR this strategy was successful on the basis that the TKDL team had, as of 2011, managed to get more than 200 patent applications based on traditional Indian medicine withdrawn or rejected (Gupta 2011; CSIR 2021). Furthermore, the mere existence of the TKDL might have had a pre-emptive effect on the misappropriation of traditional knowledge; a study conducted by the CSIR suggests that the number of patent applications based on Indian traditional medicine, filed at the EPO, had declined by 44% by 2011 (Gupta 2011). In that sense the TKDL comes across as a more systematic and efficient way to prevent misappropriation of Indian traditional medical knowledge than to merely challenge individual patents retrospectively.

Another important outcome of the TKDL is its impact on the global patent system. The TKRC is not unilaterally shaped by the IPC; the IPC has also been updated and adapted to the TKRC. Like most classification systems, the IPC is a dynamic system that is annually updated and revised to incorporate new forms of content (Kang 2012). In that regard changes to the IPC reflect developments in the body of knowledge that the patent system needs to address. Hyo Yoon Kang gives an example of how the IPC was adapted to better incorporate the recent growth of innovations within the field of chemistry, resulting in the inclusion of a new subclass for combinatorial chemistry (C40B) in the 2006 revision of the IPC (Kang 2012). Similarly, the expanding field of ethnopharmacology and the growing importance of traditional medicine for the pharmaceutical industry made traditional medical knowledge increasingly relevant for the IPC. Already before the formation of the TKDL, there was an awareness within WIPO that the IPC classification system was insufficient to document traditional knowledge, particularly regarding medicinal plants (Suwapan 2016). Consequently, in February 2001, WIPO’s IPC committee of experts appointed a Task Force on Classification of Traditional Knowledge (WIPO/GRTF/IC/2/6, § 19). In a report the following year, the task force concluded that:

the most efficient way of developing classification tools for traditional knowledge would be their integration into the IPC on the basis of its revision, in particular in the area of traditional medicine. The material for such revision could be provided by TKRC and other classification systems for traditional knowledge available in various countries. (IPC/CE/31/6, annex § 15)

Until now medicinal plants had been classified in one single subgroup, but the work of the Task Force resulted in the inclusion of a new main group called A61K 36/00 (referring to ‘Medical preparations of undetermined constitution containing material from algae, lichens, fungi or plants, or derivatives thereof, e.g. traditional herbal medicines’) in the 2003 revision of the IPC. AK61K 36/00 consists of 207 subgroups covering different categories of plants, which enable a more effective search and examination process regarding traditional medicine (Suwapan 2016).

This willingness to adapt the IPC to the TKRC jars with the common conception of international intellectual property rights regulations as a tool for a neocolonial ‘information feudalism’: a term coined by Drahos and Braithwaite and evoked by Oguamanam earlier in this text to characterize a ‘Western knowledge hegemony’ where intellectual property regimes are unilaterally imposed on the developing world by developed countries and multinational corporations (Drahos and Braithwaite 2002; Oguamanam 2008). Peter Drahos (2008, p. 153) describes how this affects the work at the patent offices in developing countries:

developing country patent offices have been integrated into a system of international patent administration, in which the grant of low quality patents by major patent offices is a daily occurrence. Developing countries for the most part have only had modest success in influencing the evolution of standards at the international level. They have little prospect of influencing the standards of patent examination in the EPO, JPO, and the USPTO, even though those standards impact on the work of their own patent offices.^1^

Seen in the context of a global information feudalism, it seems remarkable that India’s TKRC has had a direct influence on the standards of patent classification globally. Oguamanam (2008) interprets this readiness to revise the IPC to accommodate traditional knowledge as a turning point in the relation between patent law and traditional knowledge, since it discards the common assumption that intellectual property rights and traditional knowledge are inherently incompatible because they rely on different knowledge traditions. The revision of the IPC could thus imply that the TKDL not only changes how we can view and approach traditional medical knowledge, but also that the administration of patents is opening up to alternative knowledge systems.

## The Dilemmas of Documentation

When V.K. Gupta presents the TKDL as a way to bridge the gap between formal and informal knowledge systems he evokes a hierarchy where knowledge systems associated with western science are seen as more valid than those belonging to colonized subjects (Gupta 2000). This dichotomization between traditional knowledge and western science is fundamental to the international discourse on traditional knowledge protection as it underpins a wide range of extractive practices, such as acts of biopiracy where traditional knowledge is transformed into scientific innovations that can be claimed as intellectual property under the protection of patent law (Posey 2002; Oguamanam 2006; Fredriksson 2017). By translating traditional knowledge into systematically classified information that can easily be recognized as prior art, the TKDL aims to challenge this hierarchy and—as Chidi Oguamanam puts it—‘level up’ traditional medicine on a par with western medicine (Oguamanam 2008, p. 503). The question is, however, not only if a hierarchy that has been formed by centuries of colonial domination can be amended through an intervention in a classification system, but also if basing a system of traditional knowledge protection on a schematic dichotomy between traditional and western knowledge risks enforcing the very hierarchies it aims to challenge. Consequently, the TKDL has been criticized on various grounds, that can be related to its attempts to fit a diverse body of knowledge into a one-size-fits-all classification standard.

The first challenge that faces the TKDL is *what* to document. It is important to acknowledge that the TKDL does not remotely reflect the wide variety of phenomena that fall under the category of traditional knowledge. Dichotomisations between western versus traditional, or formal versus informal knowledge systems evoke a false understanding of the latter as homogenous. Traditional knowledge is rather an umbrella term that has been applied to a multitude of practices and customs ranging from traditional medicine through cultural and artistic expressions to ecological and agricultural knowledge and techniques (Perlman 2011). Indian officials readily admit that the TKDL only documents ‘a miniscule part of the codified and oral knowledge’ of India (Dhar 2017, p. 257). Not only is the TKDL limited to traditional medical knowledge, it also focuses exclusively on the four major schools of India’s traditional medicine: Ayurveda—a Hindu medical system most common in Northern India; Siddha—a medical and philosophical system with roots in Hinduism predominant in Southern India; Unani—a medical system originating from India’s Muslim tradition; and Yoga.

Different knowledge traditions also have very different social standing, ranging from very local and entirely orally transmitted expressions to widely spread, sometimes national and well documented traditions. Thomas (2010, p. 662) uses the concepts of ‘big traditions’ and ‘little traditions’ to distinguish traditional practices that are well documented, preserved, and endorsed by dominant groups in society from those that are only practised and shared among marginalized communities. While the medical knowledge included in the TKDL represents alternative medicine in a global context, it can hardly be described as marginalized in its national context and would rather fall under the category that Thomas calls ‘big traditions’. This is particularly true for Ayurvedic medicine, which has been gradually institutionalized throughout the twentieth century and is now fully juxtaposed with western medicine within India’s national health policies (Gaudillière 2014; Berger 2013). Today, Ayurvedic medicine is publicly supported and promoted through the Ministry of Ayurveda, Yoga and Naturopathy, Unani, Siddha and Homeopathy, which has also played an important role in the formation of the TKDL (Katoch, 2017).

So, while the TKDL is often framed as an initiative to protect marginalized knowledge from the global south against exploitation by the industrialized north, it primarily documents and safeguards privileged and officially promoted forms of traditional knowledge. Seemantani Sharma (2017) has criticized the TKDL for not only disregarding all traditional knowledge that is not medical (such as the vast range of traditional agricultural knowledge that exists in India), but also for privileging certain forms of traditional medical knowledge that is documented in written sources over oral traditions. In that sense the TKDL can be said to enforce a hierarchy between small traditions and big traditions, where Ayurveda, Siddha, Unani and Yoga are preserved as the national heritage of India while more socially and geographically marginalized forms of traditional knowledge remain undocumented. Furthermore, these four systems of traditional medicine are not, themselves, homogenous bodies of knowledge. While representatives of some schools within these systems have been extensively consulted, other groups of practitioners have, for various reasons, refused or been refused the opportunity to contribute to the TKDL (Reddy 2006; Thomas 2010). This implies that the database might not even reflect the full diversity of the main branches of India’s traditional medical knowledge.

Evoking a dichotomy between a formal and an informal knowledge system might be a way for Gupta to create international legitimacy for the TKDL by associating it with a global agenda on the protection of traditional knowledge from developing countries and indigenous communities against neocolonial exploitation. However, it also runs the risk of ignoring the heterogeneity of India’s traditional knowledge since the TKDL only documents a privileged part of India’s traditional knowledge that the government wants to promote (Jaffrelot 2007). As I argue elsewhere (Fredriksson, forthcoming), the TKDL might play a part in a wider Hindu nationalist agenda to protect traditional knowledge and traditional cultural expressions of Indian origin against foreign appropriation in order to claim it as part of India’s national heritage. Domestically, the TKDL can also be a tool to appropriate traditional knowledge and cultural expressions of local and indigenous communities and use it for national purposes (Fredriksson, forthcoming). This is enabled by a power dynamic where states have immensely more agency that indigenous communities in intergovernmental organizations like the UN, and are thus better positioned than indigenous communities to rely on the international community to promote their interests. This is a well-known dilemma in cultural heritage politics where UNESCO for a long time has been forced to negotiate between states and local communities who make competing claims to certain expressions of intangible cultural heritage (Blake 2019; Fredriksson forthcoming; Fredriksson 2020; Hafstein 2018; Lenzerini 2011; Lixinski 2011; Macmillan 2013).

## Classification, Knowledge Systems and Appropriation

Another challenge that faces the TKDL is *how* to document traditional knowledge within a classification system based on the traditions of western science. A wide range of scholarship has shown that classification systems are never neutral, but always impose some kind of order on the content they incorporate (Bowker and Star 1999; Mai 2011; Adler 2017; Montenegro 2019). That this has specific implications for the documentation of traditional knowledge is also well known and many have argued that classification models formed within a western knowledge tradition are inadequate to document traditional knowledge (Thomas 2010; Gebru 2015; Montenegro 2019). The following section will relate the TKDL to a body of critical research on traditional knowledge documentation and discuss the implication of such considerations for the TKDL.

Drawing on Adler (2017), Maria Montenegro describes the implementation of information standards as a ‘normalization project’ that homogenizes the information according to seemingly universal standards and erases the aspects that are significant to the communities from where it originated. Thereby, this process of normalization denies the traditional custodians the possibility to interpret the traditional knowledge according to their own norms and values (Montenegro 2019, p. 736). Thomas (2010, p. 667) argues along similar lines when he criticizes the TKDL for fixing traditional knowledge in a static form and abstracting ‘traditional practices from the larger meaning systems that suture and ground a given knowledge and practice’. Knowledge that enters the database is thus rinsed of any information that is redundant to the purpose of that particular database. This tendency to normalize and abstract knowledge is seen as particularly problematic in relation to traditional knowledge which is often defined by its holistic nature. According to Gaudillière (2014, p. 411):

The WIPO considers traditional knowledge as radically different from ‘scientific knowledge.’ Traditional knowledge is given a holistic character, and its learning is seen as relying basically on oral transmission. It is also held to be rooted in ‘communities’ (in general undefined) and in collective property systems.

Agrawal describes traditional knowledge databases as an example of ex situ preservation where the resource is taken out of its original location and stored somewhere else, which also disembeds the knowledge from that holistic context (Agrawal 2002). The process of documentation, however, does more than merely store the information out of its original context; it is also presumed to transform knowledge in a more fundamental way, as it translates it from one knowledge system into another. Reddy (2006, p. 174) describes how this is done with regard to the TKDL:

Almost 300 *Vaidyas*, Sanskrit scholars, and analysts were employed for two years to translate verses (*Slokas*) and aphorisms (*Sutras*) from the traditional pharmacopeia and Ayurvedic compendia (*Samhitas*) into structured language using a classification called the Traditional Knowledge Resource Classification; a second group isolated medicinal uses of plants from these to list them in databases; and yet another group of analysts matched these entries with original sources to compare and validate their content.

Gaudilliére (2014, p. 402) paints a similar picture when he argues that the TKDL ‘disentangles the complex composition of Ayurveda into elementary botanical units’ matching ‘the fundamental categories of botanical pharmacy’. Furthermore, Preston Hardison, who works with traditional knowledge documentation for the American first nation Tulalip Tribes, describes how the TKDL translates ‘the Sanskrit common names for species into the Linnean classification system, and from there into common names applied to the same species in other languages’ (UNEP/CBD/WG8J/4/INF/9). These descriptions of how verses and aphorisms are translated into classification codes speak to how the patent classification system’s approach to traditional medical knowledge relies on models for categorizing knowledge that are embedded in western scientific systems of classification and taxonomy dating back to Carl von Linné and his contemporaries.

If the classification of botanical knowledge draws on taxonomic systems from the Enlightenment era, then the patent classification system is a child of modernity. Eva Hemmungs Wirtén (2019) has explored how the emergence of modern patent classifications was intertwined with an early twentieth-century documentation movement, where utopian thinkers such as Paul Otlet and Henri de la Fontaine envisioned classification systems that would be comprehensive enough to organize all the world’s knowledge. On a similar note, Jens-Erik Mai (2011, p. 724) argues that the large classification systems that have emerged since the twentieth century are deeply embedded in modernity:

modern library classification has reached a point of tremendous detail orientation, and where practice has de-contextualized itself to serve only large research-oriented libraries, with an international focus, […] It has done so by focusing on rules and standards, at the expense of interpretation and locality; the overriding goal has been to create a system of global reach where everyone, everywhere uses the same system. This goal of de-contextualization, of internalization, of globalization is at the heart of modernity.

Mai continues to describe how these universalized classification systems rely on three fundamental principles: *dualism* that separates the information from the reader; *de-traditionalization* that represents information as documents independently of the contexts where they are used and (re)produced; and *globalization* which alienates information from its location in time and space (Mai 2011).

While Mai discusses modern classification systems in general, Agrawal describes a similar, three-step process with respect to the documentation and ex situ preservation of traditional knowledge. The initial step involves singling out specific aspects of an indigenous culture that can potentially be developed and utilized, and separating those from their cultural context (Agrawal 2002, p. 290). Agrawal calls this *particularization*. This is followed by a step of *validation* of the traditional knowledge against the norms of western science and finally a moment of *generalization* where the information is made useful in a wider context. Both for Mai and for Agrawal, the consequences are the same: that the dominant principles of classification alienate the information from its original users, decontextualize it from its original context and strip it from all meaning that is not relevant to or compatible with the logics and purpose of the classification system.

Reddy (2006, p. 165) gives a perspective on how this could apply to the TKDL when he argues that ‘making Ayurvedic heritage legible’ through the translation of verses and aphorisms into patent classifications ‘involves a shift from safeguarding traditional knowledge to the documentation of that knowledge *as* information’. This transformation of traditional knowledge into information locates the documented knowledge ‘somewhere on a continuum between “wisdom” and “data,” a process that transforms the very nature of these cultural objects in question’. Reddy argues that traditional knowledge is subjected to a homogenizing process ‘that strips away all the detailed, contextual aspects that could even potentially mark it as indigenous’ (Reddy 2006, p. 175).

The question is, however, to what extent theories that approach traditional knowledge databases as a form of ex situ preservation are applicable to the TKDL. WIPO makes a distinction between two different kinds of protective databases, aiming to provide either *positive* or *defensive* protection. Positive protection refers to initiatives to save and preserve traditional knowledge for posteriority and promote the traditional owners’ control and use of that knowledge. Defensive protection on the other hand refers to databases whose primary intention is to protect knowledge against misappropriation (WIPO/GRTKF/IC/3/6). One example of a database providing positive protection often evoked by WIPO is StoryBase, which was constructed by the already mentioned Tulalip Tribes around the same time as the TKDL. StoryBase collects traditional ecological knowledge that has been maintained by the Tulalip community for centuries and stores it for future generations (WIPO/GRTKF/IC//3/6). This database is managed by a local indigenous community to preserve a part of their cultural heritage which is at risk of going extinct, and its content is primarily reserved for use and dissemination within that community.

The TKDL, on the other hand, is a strictly defensive database that is not designed to store and preserve knowledge as heritage but to limit the possibilities to patent that knowledge. This merely requires the database to identify traditional knowledge as previous art by providing metadata pointing to the existence and location of that knowledge. It is miselading to discuss the TKDL as a form of ex situ preservation of traditional knowledge since it does not take the knowledge out of its original context, and while it might be said to transform ‘wisdom’ to ‘data’ it does not replace that wisdom with data. The TKDL rather adds a layer of metadata (classified in the TKRC) that makes the protected knowledge more accessible according to systematic principles tailored for international patent examiners. Bowker and Star’s (1999, p. 61) phrasing that classification systems ‘reflect’ the social world thus applies particularly well to the TKDL since its major accomplishment is to catalogue metadata that reflects knowledge that already exist as living practices across India and is well documented in various socurces.

While the protection of traditional knowledge is indeed entrenched in a cultural heritage discourse, and the TKDL is often articulated as a protection of India’s cultural heritage against foreign exploitation, it is fundamentally dictated by economic concerns rather than by cultural heritage considerations. Essentially, the TKDL regulates the commercial exploitation of traditional knowledge resources of Indian origin. When he expands on the notion of how the TKDL ‘levels up’ traditional knowledge in relation to western science, Oguamanam (2008, p. 503) acknowledges that a classification system like the TKDL comes nowhere close to adressing the fundamental property relations that underpin biopiracy and other forms of misappropriation:

TKDL calibrates, or levels up, traditional medicine with its Western counterpart. This creates a semblance of psychological parity in favor of traditional medicinal knowledge vis-à-vis the extant recognition of Western scientific medicine under the patent regime. The difference is that while traditional medicine-related knowledge may be, at least in theory, freely accessible because it is part of the public domain, its Western biomedical or scientific counterpart is protected for the term of any applicable patent.

This indicates that even when interventions in classification systems suceed in alleviating the hierarchies between different knowledge systems, they do not interfere with fundamental property relations. Challenging the patent system is however not the goal of the CSIR. The next section will discuss how the TKDL not only protects traditional knowledge against commercial exploitation abroad, but plays a part in a wider strategy to make that knowledge more accessible for exploitation domestically.

## Ayurveda and India’s alternative modernity

Bearing the discussion in the previous section in mind, it is important not to resort to a simplistic polarization between two homogenous and mutually exclusive knowledge systems. Critical scholarship on documentation and traditional knowledge would argue that traditional knowledge databases based on established western classification models, like that of the IPC, incorporate traditional knowledge within a modern knowledge and classification system. While this, on the one hand, presents the TKDL as an attempt to ‘level up’ traditional knowledge compared to western science, it can also imply that the TKDL subsumes the knowledge system of traditional knowledge under the modern patent classification system, and thus further enforces the hierarchies it seeks to amend. Uncritically relying on a polarization between two knowledge systems, however, risks homogenizing the rich variety of traditional knowledge and enforcing an arbitrary dichotomy between modern western science and ancient eastern knowledge, where the former is seen as progessive and dynamic while the latter becomes static wisdom that needs to be preserved in a state of origin.

A more nuanced approach to make sense of a heterogenous range of knowledge would be to see dichotomies like written versus oral, formal versus informal, scientific versus traditional knowledge not as fixed and mutually exclusive categories but as ambigous and constantly transforming positions on interacting and overlapping scales. These categories are not essential, but rather functional in the sense that they are attributed and employed differently to serve certain strategic pruposes. Historically we have seen this in colonial strategies to create hierarchies that legitimize expolitation of resources from the global south. In its attempts to challenge the hierarchy between formal and informal knowledge systems the TKDL also employs the dichotomy between western and traditional knowledge systems to gain political legitimacy in the international community. The cost of that is however that it risks reinforcing colonial dichotomies.

Analyzing the TKDL’s role in its national, Indian, context however gives another perspective that begins to undermine those dichotomies. As already mentioned, traditional Indian medicine in general and Ayurveda in particular are dynamic knowledge practices that constitute national, publicly supported health systems that have been continuously modernized throughout the twentieth century (Berger 2013). Readymade and commercially produced Ayurvedic medicine has existed since the 1930s, but the production of traditional Indian pharmaceuticals entered a new phase in the 2000s. In this period both the mode and scale of production has become increasingly industrialized to the extent that some Indian Ayurvedic drug companies now operate internationally (Gaudillière 2014). In a study of the development of the Ayurvedic drug industry since the 1980s, Pordié and Gaudillière (2014) describe how Ayurvedic medicine in postcolonial India has undergone an alternative modernization, which they characterize as ‘the reformulation regime’. This refers to a systematic reformulation of traditional Ayurvedic drugs into new compositions. These reformulated compositions have the benefit that they can more easily be traded on a national and international market, but also protected as intellectual property since they are, unlike traditional medicine, not prior art. These so-called ‘Ayurvedic proprietary medicines’ (Pordié and Gaudillière 2014, p. 64), are pharmacologically different from both traditional Ayurvedic medicine and from western biomedicine, and represent a form of alternative modernity in Indian medicine that bridges the gap between traditional medicine and modern science.

The reformulation regime works in three steps. first it homogenizes and standardizes the preparation of the drugs; then it simplifies the formulations to be better suited for mass production; and finally it adapts the formulations to a global market. This largely corresponds to the steps of ‘particularization’, ‘validation’ and ‘generalization’ that Agraval connects with classification of traditional knowledge. In that sense, the transformation that western regimes of knowledge and property are often accused of imposing on traditional knowledge has already been enforced by India’s domestic drug industry with regards to Ayurvedic traditional medicine. This preexisting local context is crucial for understanding the role of the TKDL.

At a seminar with the World Health Organization in 2005, V.K. Gupta described how the TKDL serves to connect India’s different knowledge systems to each other in a more structured way. He argues that while Ayurveda, Siddha and Unani have existed independently of each other for centuries ‘TKDL is a mechanism, which can validate these systems against each other and likely enhance active research based on TK [traditional knowledge] through reverse pharmacology by [one] order of magnitude.’ (Gaudillière 2014, p. 404). Here TKDL comes across not only as a protection against foreign appropriation, but also as a means to document India’s traditional medical systems in a way that makes that knowledge more manageable, and exploitable. Gaudillière (2014, p. 404) concludes:

This brings in a second, less visible dimension of the TKDL, which goes hand in hand with the industrial pharmaceuticalization of Indian traditional medicines. In practice, the TKDL has created a vast unified corpus, which, because of its digital nature (and in spite of its heterogeneity), can easily be mined, that is, for specific plants, for specific diseases or symptoms, and for correlations between the botanical and the medical.

Seen in its national context and history, TKDL is not merely an adaptation to standards imposed by WIPO and the international patent regime, but also part of a long-term domestic strategy to modernize and systematically utilize traditional medical knowledge as a national resource. Elsewhere (Fredriksson, forthcoming) I have argued that while the TKDL presents itself to the international community as an initiative to protect traditional knowledge from the developing world against neocolonial exploitation, it also creates an information infrastructure that helps the CSIR to develop and patent innovations based on traditional medical knowledge. Thus, while the TKDL prevents the commodification of Indian traditional medicine by foreign actors it also promotes domestic uses of such knowledge to social and economic benefits of the nation.

India’s attempts to modernize Ayurvedic medicine does not disapprove the assumption that there are different knowledge systems that are differently positioned in a postcolonial order of power. It does, however, challenge the simple binarity between two distinctively different knowledge systems suggested, for instance, by V.K. Gupta. Firstly, the modernisation of India’s traditional medicine that predates the formation of the TKDL examplify that most schools of traditional medical knowledge are heterogenous, dynamic and continously evolving systems of knowledge. Secondly, this points to the arbitrary nature of the very distinction between traditional knowledge and modern science. Finally, the framing of the TKDL implies that the protection of traditional knowledge can serve many different purposes and interests. When presented to WIPO, the TKDL is contextualized within an international discourse about protecting indigenous and traditional knowledge of the developing world against neocolonial exploitation; in a domestic context it rather fits into a national strategy to document and modernize traditional knowledge to the benefit of social and economic development.

## Conclusions

Looking at the TKDL as an intervention against the misappropriation of traditional knowledge reveals both possibilities and pitfalls with using databases as a means to protect traditional knowledge. On the one hand the TKDL shows that a well designed documentation and classification system can bridge the presumed gap between formal and informal knowledge systems, acknowledge traditional knowledge as prior art and thereby prevent misappropriation of such knowledge. Furthermore the revisions made to the IPC to include traditional medical knowledge suggest that the global patent system and its institutions are open to adapting their documentation standards to include content from non-western knowledge traditions.

On the other hand, the TKDL also highlights dilemmas with traditional knowledge databases, as well as with basing traditional knowledge protection on too simplistic dichotomies between traditional and western knowledge. One dilemma concerns how the selection of what to document is dictated by national interests or international political agendas whose priority is not necessarily the rights of local communities. This becomes evident in how the TKDL only includes knowledge from major and well documented schools of traditional medicine that are practised and promoted by dominant groups in society. Furthermore, critical documentation studies often argue that documentation transforms the information it catalogues, translates it from one knowledge system to another and strips traditional knowledge of the values and connotations that are most important to its original custodians. It can however be questioned to what extent this applies to a database like the TKDL which is not an ex situ depository of traditional knowledge, but rather a catalogue of metadata intended to regulate the commercalization of that knowledge.

The international discourse on traditional knowledge protection tends to address the subjects of colonial oppression as a homogenous group and see the interests of indigenous communities and postcolonial states as uniform. In its interactions with the international community, India benefits from this convergence and draws on how biopiracy, with Oguamanam’s (2008, p. 32) words, has become ‘a touchstone for solidarity among indigenous peoples and former colonies of the south […] in their resistance to Western knowledge hegemony’. Promoting the TKDL as part of a international struggle against neocolonial appropriation and exploitation of traditional knowledge from the global south creates international legitimacy for the TKDL as a social justice tool, but masquerades its role in promoting national, economic interests.

To conclude, the construction of the TKDL and its framing within a national and an international discourse calls into question a number of assumptions about the documentation of traditional knowledge. Seen against the backdrop of India’s strategic modernization of Ayurvedic medicine in the late twentieth century, the construction of the TKDL challenges the dichotomization of western and non-western knowledge systems that tends to underline not only colonial discourses but also critical theories and strategies aiming to challenge colonial stereotypes. Finally, the different ways to approach and conceptualize the TKDL reminds us that databases, too, are heterogenous and multidimensional. Not only do different kinds of databases, such as defensive catalagues of metadata like the TKDL or positive ex situ depositories of knowledge like StoryBase, follow different dynamics; each database can also play disparate roles and serve various purposes in different political contexts.

## Footnotes

1 Japan Patent Office (JPO) and United States Patent and Trademark Office (USPTO).
